# Proteasome caspase-like activity regulates stress granules and proteasome condensates

**DOI:** 10.3389/fcell.2025.1570499

**Published:** 2025-06-05

**Authors:** Shirel Steinberger, Julia Adler, Nadav Myers, Yosef Shaul

**Affiliations:** Department of Molecular Genetics, Weizmann Institute of Science, Rehovot, Israel

**Keywords:** stress granules, proteasome condensates, proteasome caspase-like activity, intrinsically disordered proteins, 20S proteasome

## Abstract

The 20S proteasome maintains cellular protein homeostasis, particularly during stress responses. In a previous study, we identified numerous 20S proteasome substrates through mass spectrometry analysis of peptides generated from cellular extracts degraded by purified 20S proteasome. Many substrates were found to be components of liquid-phase separation, such as stress granules (SGs). Here, we demonstrate the degradation products arise from the caspase-like (CL) proteasomal activity. To investigate the functional implications of CL activity, we generated cell lines devoid of CL function by introducing the PSMB6 T35A mutation. These mutant cells exhibited slower growth rates, heightened sensitivity to stress, and activation of the unfolded protein response (UPR), as indicated by elevated levels of spliced XBP1 (sXBP1) and stress markers. Cells were subjected to arsenite and osmotic stress to assess their responses. Our findings reveal that CL activity is crucial for efficient SG assembly but does not significantly affect SG clearance. Interestingly, in these mutant cells, proteasomes were more cytoplasmic under normal conditions but formed nuclear condensates/granules (PGs) upon NaCl osmotic stress. However, the PGs were unstable and rapidly dispersed. These findings underscore the important role of the proteasome’s CL activity in managing stress-induced dynamics of liquid-liquid phase, highlighting its importance in cellular adaptation to proteotoxic and genotoxic stress conditions.

## 1 Introduction

The 26S proteasome is essential for cell survival and proliferation, playing a key role in regulating cellular protein quality control (Naujokat and Hoffmann, 2002; Collins and Goldberg, 2017; Coux et al., 1996). It removes damaged, misfolded, or unneeded proteins to maintain cellular homeostasis and prevent the accumulation of toxic aggregates (Pohl and Dikic, 2019). This process is critical for various cellular functions, including cell cycle regulation, immune response, and stress adaptation. At the core of the 26S proteasome complex lies the 20S proteasome, which serves as its central component. The 20S proteasome is composed of four stacked rings, forming a cylindrical shape with a central channel where proteolysis occurs. These rings are made up of 28 protein subunits organized as follows: Two outer rings consist of seven α or PSMA subunits. Two inner rings consist of seven PSMB or β subunits (Groll et al., 1997). The PSMB subunits form the proteolytic chamber, where the actual degradation of proteins takes place. Three of the PSMB subunits are catalytically active and possess protease activity, each with distinct specificities: PSMB6 (**β1)** exhibits CL (or peptidyl-glutamyl peptide hydrolyzing) activity, cleaving after acidic residues. PSMB7 (β2**)** exhibits trypsin-like activity, cleaving after basic residues. PSMB5 (**β5)** exhibits chymotrypsin-like activity, cleaving after hydrophobic residues (Kisselev et al., 1999a; Kim et al., 2018; Groll et al., 1999). The chymotrypsin-like activity is generally considered the most crucial for its function (Kisselev et al., 2006). An important question is whether each of the three types of proteolytic activities of the 20S catalytic particle has a unique role. Here, we addressed this question by generating cell lines devoid of CL activity.

We investigated the role of CL activity in the formation of stress granules (SGs) and proteasome granules. SGs are cytoplasmic, membraneless organelles formed via liquid–liquid phase separation (LLPS) in response to stressors like heat shock, oxidative stress, or carbon deprivation, driven by interactions involving RNA-binding proteins like G3BP (Protter and Parker, 2016; Guillén-Boixet et al., 2020; Yang et al., 2020; Gwon et al., 2021). Containing untranslated mRNAs, RNA-binding proteins, translation factors, and signaling components, SGs regulate RNA metabolism and stress signaling (Hofmann et al., 2021; Ries et al., 2019). Their formation is primarily controlled by eIF2α phosphorylation and RNA-binding proteins, which mediate nucleation and stabilization (Kedersha et al., 1999; Millar et al., 2023).

SG dynamics depend on the balance between aggregation and disaggregation, influenced by chaperones (e.g., Hsp70, Hsp90) (Mediani et al., 2021), post-translational modifications (e.g., phosphorylation, ubiquitination, methylation) (Wang et al., 2023), and remodeling enzymes (e.g., USP10, DDX3) (Buchan, 2024). Disassembly, driven by eIF2α dephosphorylation, translation restoration, and disaggregase activity, occurs when stress resolves, restoring normal cellular functions. Dysregulated SG dynamics are linked to diseases like cancer, viral infections, and neurodegeneration (Li et al., 2013; Markmiller et al., 2018).

The role of proteasomes in SG assembly and dissolution is not well understood. Inhibition of the Ubiquitin-Proteasome System (UPS) induces SGs Formation (Mazroui et al., 2007). ZFAND1 is a regulator of SG clearance, that interacts with the 26S proteasome and p97 (valosin containing protein (VCP), a chaperone like protein), which are recruited to arsenite-induced SGs for their dissolution (Turakhiya et al., 2018). Proteasome inhibitors also indirectly might regulate SG formation through certain cellular pathways, mRNA metabolism, cellular stress responses, and protein quality control systems. However, whether and which of the proteasomal different proteolytic activities are directly involved in SG formation and dissolution awaits further investigation.

Under certain conditions, proteasome condensates, referred to as proteasome granules (PGs), exhibiting properties of liquid-liquid phase separation (LLPS), are formed preferentially in the nuclei of the animal cells (Yasuda et al., 2020; Steinberger et al., 2023). Some of the essential components of PGs are p97 (VCP), RAD23B, a substrate-shuttling factor for the proteasome, and UBE3A, ubiquitin-protein ligase E3A also known as E6AP (Yasuda et al., 2020). Mechanistically, LLPS assembly is mediated via multivalent interactions of two ubiquitin-associated domains of RAD23B and ubiquitin chains consisting of four or more ubiquitin molecules (Yasuda et al., 2020).

In yeast, proteasome condensate, also known as proteasome storage granules (PSGs), are formed under certain stress conditions when reaching stationary phase growth or nutrient deprivation (Laporte et al., 2008; Gu et al., 2017; Waite et al., 2024). Unlike the nuclear PG in animal cells, the PSGs are cytosolic (Laporte et al., 2008). Mechanistically, PSGs contain ubiquitin chains together with Rad23 and Dsk2, the proteasome shuttle factors, which are critical component for PSG formation (Waite et al., 2024), highlighting the mechanistic similarity between PGs and PSGs.

When the cells were treated with the proteasome inhibitor MG-132, which preferentially inhibits the chymotrypsin-like (β5) activity of the proteasome, but it also inhibits the caspase-like (β1) and trypsin-like (β2) sites to a lesser extent, or b-AP15, a specific inhibitor of the deubiquitinating enzymes, the number and size of the PGs increased, and the rate of clearance was significantly delayed (Yasuda et al., 2020). However, the importance of proteasome CL activity in PG formation remained an open question. Here, we report the generation of cell lines with inactivated proteasome CL and demonstrate that they are inferior in the level of SGs and PGs formation under stress.

## 2 Materials and methods

### 2.1 Materials

Sodium (meta)arsenite, doxorubicin, antimycin A, rotenone, poly-D-lysine hydrobromide were purchased from Sigma-Aldrich (Burlington, MA, United States); Hoechst33342 from Molecular Probes (Eugene, OE, United States); bortezomib from APExBIO (Houston, TX, United States). Cisplatin was from ABIC (Israel).

### 2.2 Cells and cell culture

We have previously edited HEK293 (ATCC, CRL-1573 ^™^) to establish the PSMB6 T35A HEK293 mutant cell with reduced or abolished proteasome CL activity (Reuven et al., 2021). We used these cell lines to introduce the YFP tag to PSMB6 and PSMB6 T35A genes, using CRISPR technology, as previously described (Reuven et al., 2019; Reuven et al., 2021). The YFP tag was inserted only in one of the alleles. Cell lines were also labeled with the stress granules marker, G3BP1-mCherry. G3BP1-mCherry was introduced via lentiviral transduction and may have integrated randomly into the genome, as described elsewhere (Steinberger et al., 2023). Cells were grown at 37 °C in a humidified incubator with 5.6% CO2 in Dulbecco’s modified Eagle’s medium (DMEM; GIBCO, Life Technologies, Thermo Scientific, Waltham, MA, United States) supplemented with 8% fetal bovine serum (GIBCO), 100 units/mL penicillin, and 100 μg/mL streptomycin (Biological Industries, Beit Hemek, Israel). The Incucyte® SX1 Live-Cell Analysis System (Sartorius) was used to continuously image and analyze live cells within a standard tissue culture incubator. Cells were plated at 14,000 cells per well in 24-well plates, imaged every 2 h at ×10 magnification with 25 images per well, and analyzed using the system’s software to calculate percent confluence. Cell doubling time was determined by plotting the log2 of confluence over time and calculating the slope (Reuven et al., 2021).

### 2.3 Live cell imaging

Cells were seeded on a 96-well glass bottom Microwell plate 630 µL black 17 mm low glass from Matrical Bioscience (MGB096-1-2-LG), coated with poly-D-lysine hydrobromide, and were allowed to adhere overnight. Before the imaging, cell media were supplemented with 5 µM Hoechst 33342. The plate was placed in the microscope chamber, and cells were maintained at 37°C and 5% CO_2_ for the duration of the experiment. Osmotic stress was induced by replacing the medium at time zero with medium supplemented with 150 mM NaCl or 200 mM sucrose. To induce proteotoxic stress, sodium (meta)arsenite was added to the medium to a final concentration of 1 mM. Images of four different sites in a well were taken every 4 min for 3 hours using a VisiScope Confocal Cell Explorer live cell imaging system with a ×60-oil objective. Data was processed and analyzed as described in (Steinberger et al., 2023). Statistical tests of the two-tailed t-test were performed to assess significance using Excel.

### 2.4 Immunoblot analysis

SDS-PAGE and immunoblotting were performed as previously described (Levy et al., 2007) using RIPA buffer (50 mM Tris-HCl pH 7.5, 150 mM NaCl, 1% Nonidet P-40 (v/v), 0.5% deoxycholate (v/v), 0.1% SDS (w/v)) supplemented with a cocktail of protease inhibitors (APExBIO, Houston, TX) for cell extract preparation. Antibodies used in this study: pS52 eIF2α (Thermo Scientific, Rockford, Il, United States), ATF4 (Cell Signaling, Beverly, MA, United States), eIF2α, and hsp90 (Santa Cruz Biotechnology, Santa Cruz, CA, United States). Horseradish peroxidase-conjugated secondary antibodies were from Jackson ImmunoResearch Laboratories, West Grove, PA. Enhanced chemiluminescence was performed with the EZ-ECL kit (Biological Industries, Beit Hemek, Israel), and signals were detected by the ImageQuant LAS 4000 (GE Healthcare, Piscataway, NJ, United States).

*20S Proteasome degradation.* Dataset used for analysis - PXD010132 from the PRIDE (*), https://www.ebi.ac.uk/pride/, MS proteomics repository described in (Myers et al., 2018).

### 2.5 Protein analysis

20S Proteasome degradation: Dataset used for analysis - PXD010132 from the PRIDE (Vizcaíno et al., 2016), https://www.ebi.ac.uk/pride/, MS proteomics repository described in (Myers et al., 2018).

All protein sequences were retrieved from the UniProt database (http://www.uniprot.org/) using the curated Swiss-Prot knowledgebase (Consortium, 2012). Statistical analyses and data visualization were conducted using MATLAB 2016b (The MathWorks, Natick, 2014). For hypothesis testing, p-values were calculated using a two-sided non-parametric Wilcoxon rank-sum test applied to continuous data. While all data points were included in the statistical analyses, outliers were excluded from box plots to ensure clarity and improve visualization.

To identify amino acid preferences around the cleavage site in 20S core particle (CP) substrates (Wolf-Levy et al., 2018) peptide sequences were aligned to their corresponding host protein sequences to extract the four amino acids upstream and downstream of each cleavage site (positions P4–P4’ from both the N- and C-termini of the peptides). Frequency profiles were generated using IceLogo (Colaert et al., 2009), a tool that highlights statistically over- or under-represented amino acids, thus revealing sequence motifs associated with proteolytic cleavage. Of all cleavage sites identified, the remaining 749 cleavage sites could be clustered using a distance function based on a substitution matrix focusing on amino acid positions P1 and P2 (Wolf-Levy et al., 2018). The number of clusters was set to three, corresponding to the three distinct catalytic activities of the proteasome.

### 2.6 RNA extraction, cDNA preparation and analysis

Total RNA was extracted using TRI Reagent (MRC, Beverly Hills, CA, United States). First-strand cDNA synthesis was performed using the iScript cDNA Synthesis Kit (Quanta, Houston, TX, United States). Quantitative real-time PCR (qRT-PCR) was conducted using the LightCycler 480 (Roche, Basel, Switzerland) with PerfeCta® SYBR Green FastMix (Quanta). All qPCR results were normalized to TBP1 mRNA levels.

### 2.7 Statistical analysis

The statistical significance (*p*-value) between means was assessed by two-tailed Student’s *t*-tests.

## 3 Results

### 3.1 Caspase-like is active in substrate degradation by 20S proteasome *in vitro*

**FIGURE 1:**
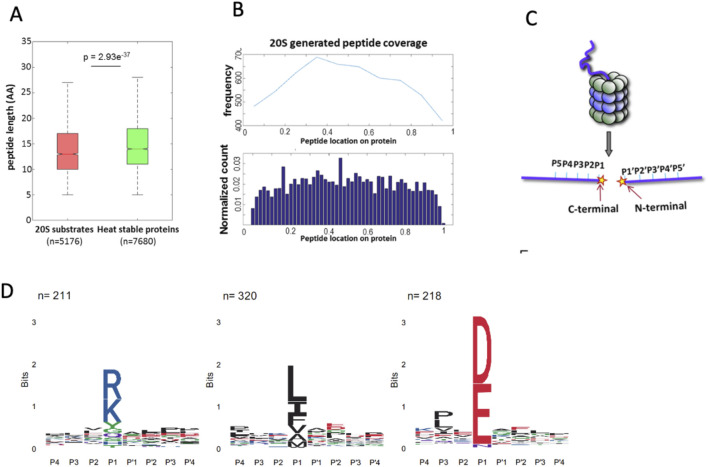
Characterization of 20S proteasome substrates and cleavage specificity. **(A)** Box plot comparing the peptide lengths of 20S proteasome-generated products (red, n = 5176) with those derived from trypsin digestion of heat-stable proteins (green, n = 7680). The 20S proteasome generates shorter peptides on average (p = 2.93e−37). **(B)** Distribution of peptide locations across substrate proteins as determined by mass spectrometry. Protein sizes were normalized from 0 to 1 to allow comparison across proteins of varying lengths. Peptides were mapped based on the position of their N-terminal residue, which was used to localize them along the normalized protein length. The upper panel shows the frequency of peptides spanning the entire substrate, while the lower panel displays the normalized count of peptide locations, indicating uniform degradation without specific cleavage preferences. **(C)** Schematic representation of substrate positioning within the proteolytic chamber of the 20S proteasome. The regions adjacent to the cleavage site, P5–P1 on the C-terminal side and P1′–P5′ on the N-terminal side, influence cleavage efficiency and specificity. **(D)** Sequence preferences of the 20S proteasome cleavage sites, illustrated by IceLogo analysis. Peptides digested by proteasomes *in vitro* clustered to acidic, basic, and hydrophobic residues in the cutting site. The y-axis labeled in “bits” reflects the information content at each position in the sequence logo.

In previous experiments, we incubated heat-stable proteins derived from cell extracts enriched for intrinsically disordered proteins and protein regions (IDPs/IDRs) with purified 20S proteasomes to identify potential 20S substrates (Myers et al., 2018). Proteasomes are known for their length-specific degradation, predominantly generating oligopeptides ranging from 3 to 25 amino acid residues (Kisselev et al., 1999b; Voges et al., 1999; Groll and Clausen, 2003). We compared the sizes of the oligopeptides produced by the 20S proteasome with those generated by trypsin digestion of the heat-stable proteins. Notably, the 20S proteasome produced shorter peptides compared to trypsin (Figure 1A). Further analysis of the mass spectrometry (MS) data revealed that the peptides generated by the 20S proteasome spanned the entire length of the substrate proteins. This suggests that the 20S proteasome does not exhibit a specific preference for cleavage sites, as the degradation occurs uniformly across the substrate (Figure 1B).

The structural architecture of the 20S proteasome proteolytic chamber plays a crucial role in substrate specificity. Binding pockets surrounding the active sites determine substrate affinity, guiding the interaction between substrates and the proteasome active sites. Consequently, the amino acid sequence around the scissile bond significantly influences degradation efficiency and specificity (Groll and Clausen, 2003; Huber et al., 2012). The amino acid positions immediately preceding and following the cleavage site are illustrated (Figure 1C). To gain a deeper understanding of the sequence preferences of 20S proteasome substrates, we analyzed the amino acid composition at the N-terminal (P1′–P5′) and C-terminal (P5–P1) regions of the peptides generated by the 20S proteasome. This analysis was performed using our peptide database derived from 20S proteasome-generated products (Myers et al., 2018). The IceLogo software (Colaert et al., 2009) was used to generate a frequency profile that highlights amino acid preferences or disfavor surrounding the cleavage site in 20S proteasome substrates. This analysis reveals a distinct degradation sequence characteristic of 20S proteasome activity (Figure 1D). Right Logo (D, E) is the sequence signature of the proteasome CL activity. These findings suggest that the 20S proteasome CL is active in the degradation of the IDPs/IDRs enriched substrates.

### 3.2 Proteasome CL activity deficiency impairs cell growth, disrupts stress responses, and activates the unfolded protein response (UPR)

**FIGURE 2:**
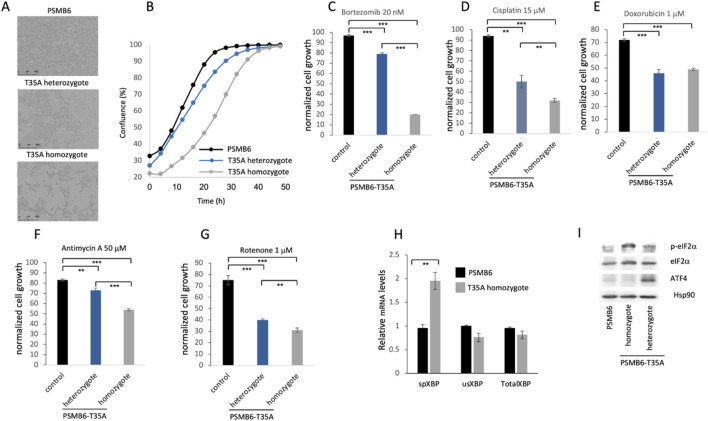
Effects of PSMB6 T35A mutation on cell growth, stress response, and unfolded protein response (UPR). **(A)** Representative phase-contrast images of wild-type PSMB6, heterozygous, and homozygous PSMB6 T35A mutant cells, showing slower growth in the mutants. **(B)** Growth curves of PSMB6 wild-type, heterozygous, and homozygous T35A mutant cells over 50 h, using Incucyte® SX1 live-cell analysis system. A significantly reduced rate of confluence achievement in mutant cells, particularly in homozygotes, is shown. **(C–E)** Growth rates of PSMB6 T35A mutant and wild-type cells treated with bortezomib, an inhibitor of proteasome chymotrypsin-like activity **(C)**, cisplatin, a DNA-damaging agent **(D)**, or doxorubicin, a DNA-intercalating agent **(E)**. Normalized cell growth was calculated by comparing each treated cell line to its respective untreated control. Homozygous mutants show severe growth inhibition upon treatment. **(F–G)** Effect of mitochondrial inhibitors, antimycin A **(F)** and rotenone **(G)**, on cell growth. **(H)** Relative levels of spliced XBP-1 (sXBP-1), unspliced XBP-1 (usXBP-1), and total XBP-1 mRNA were measured by qRT-PCR. Spliced XBP-1 levels are elevated in homozygous PSMB6 T35A cells, indicating UPR activation. **(I)** Immunoblot analysis of UPR markers (p-eIF2α and ATF4) in PSMB6 wild-type and T35A mutants. Homozygous PSMB6 T35A cells exhibit elevated levels of p-eIF2α and ATF4. Hsp90 is used as a loading control to normalize protein expression. In each case **(C–H)** the results are of 3 independent experiments.  **p < 0.01,  ***p < 0.001.

To evaluate the role of CL activity in the cells, we generated both heterozygote and homozygote PSMB6 T35A edited cell lines, demonstrating partial or lack of CL activity, respectively, as previously described (Reuven et al., 2021). The growth rate of the mutants was much slower, especially in the PSMB6 T35A homozygote cells (Figures 2A,B). Next, we treated the cells with bortezomib, an inhibitor of proteasome chymotrypsin-like activity, and the growth of homozygote cells was severely compromised (Figure 2C). The edited cells show a much lower growth rate when treated with cisplatin, a DNA damage agent (Figure 2D), and doxorubicin, a DNA intercalating agent (Figure 2E). Inhibition of cellular respiratory by each Antimycin A and rotenone reduced the growth of the PSMB6 T35A edited cells (Figures 2F,G). Given the important role of proteasome in reducing ER stress and unfolded protein response (UPR), we asked whether cells deficient in CL activity induce the UPR. To this end, the levels of some of the UPR markers were measured (Read and Schröder, 2021). The level of spliced XBP-1 (sXBP-1) mRNA was increased in the homozygote PSMB6 T35A edited cells (Figure 2H). ATF4 and p-eIF2α levels were analyzed by immunoblot and showed significant induction in the PSMB6 T35A edited cells (Figure 2I). The T35A mutation in PSMB6, generating proteasomes deficient in CL activity, appears to enhance the activation of the UPR, as evidenced by increased spliced XBP1 mRNA and elevated stress markers. These data demonstrate that PSMB6 T35A mutation, impairing CL activity, slows cell growth and sensitizes cells to proteotoxic and genotoxic stress. The mutation also induces UPR activation.

### 3.3 Proteasome caspase-like activity regulates stress granules

**FIGURE 3:**
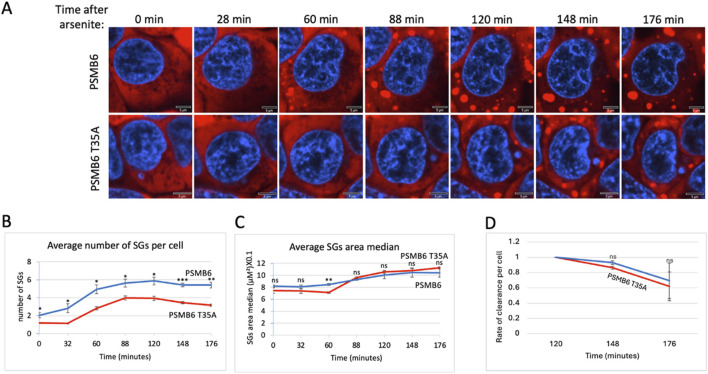
SGs dynamic over time after arsenite treatment in wild-type and PSMB6 T35A mutant cells. **(A)** Shows fluorescent images of cells at various time points (0–176 min) after arsenite treatment. Blue represents nuclear staining (DAPI). Red represents G3BP-mCherry expression. The scale bar represents 5 µm. In PSMB6 wild-type cells, SG formation appears robust and increases over time after arsenite treatment. In PSMB6 T35A mutant cells, SG formation is reduced or delayed, with fewer SGs evident compared to the wild-type cells. **(B)** The average number of stress granules per cell over time. Note that SGs form rapidly in PSMB6 wild-type cells, reaching a peak between 88 and 120 min. Meanwhile, in PSMB6 T35A mutant cells, SG formation is significantly reduced at most time points, as indicated by statistical markers. **(C)** SG area median per cell over time. **(D)** Rate of clearance of SG per cell along the experiment. The curves were calculated based on data obtained from quantifying 3-4 frames of 1,024 × 1,024 pixels. Each experiment was repeated at least twice. The data were analyzed by 2-tailed unpaired t-test  *p < 0.05,  **p < 0.01,  ***p < 0.001.

Previously, we reported that the purified 20S proteasome degrades proteins associated with stress granules (Myers et al., 2018). As shown above (Figure 1D), CL activity was the primary factor in degrading these proteins. This prompted us to investigate whether proteasome CL activity regulates stress granule (SG) assembly. To this end, cells expressing G3BP-mCherry, a marker of SGs, were treated with arsenite, a well-known inducer of SG formation. Fluorescent imaging over time (0–176 min) showed robust SG assembly in PSMB6 wild-type cells, with the number of SGs gradually increasing up to 88–120 min before slowly dispersing (Figure 3A). By contrast, PSMB6 T35A mutant cells, devoid of CL activity, exhibited significantly fewer SGs per cell (Figures 3A,B). Interestingly, despite the reduced number of SGs in mutant cells, their size was comparable to those in wild-type cells (Figure 3C).

The median SG area per cell was similar between the 2 cell types, suggesting that CL activity primarily impacts SG number rather than size. The relatively parallel curves for SG clearance in both cell types suggest that the dynamics of SG rate of clearance is similar, regardless of CL activity (Figure 3D). However, given the modest differences observed and the low number of granules per cell, this interpretation should be treated with caution. Additionally, we cannot exclude the possibility that differences in granule structure or compactness—parameters not directly assessed here—may influence their clearance. Together, these results highlight the proteasome’s critical role in regulating SG assembly during arsenite-induced proteotoxic and oxidative stress responses.

### 3.4 Proteasome caspase-like activity regulates SG dynamics under osmotic stress

**FIGURE 4:**
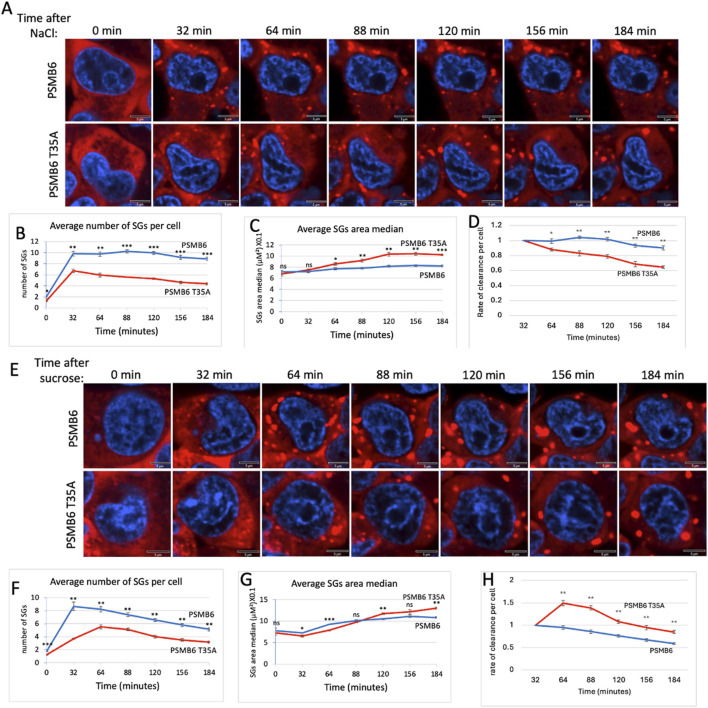
SG dynamic over time in wild-type and PSMB6 T35A mutant cells after osmotic stress. **(A)** Microscopic images of wild-type G3BP-mCherry HEK293 (PSMB6) and PSMB6 T35A mutant cells at the indicated time points. Blue represents nuclear staining (DAPI). Red represents G3BP-mCherry expression. Stained for stress granules (red) and nuclei (blue) at different time points. SGs are increased after NaCl treatment in both cell types, but their formation appears more pronounced or persists longer in the wild-type condition compared to the mutant. **(B)** The average number of stress granules per cell at different time points. The blue line (wild-type) shows a higher and sustained number of SGs over time. The red line (mutant) shows a lower peak and faster reduction in SG numbers. **(C)** As in panel B but the average SGs area median over time for both cell types shown. **(D)** Rate of cearance of SG per cell along the NaCl treatment. **(E)** Similar to panel A but cells were treated with sucrose. **(F)** Similar to panel B but cells were treated with sucrose. **(G)** Similar to panel C but cells were treated with sucrose. **(H)** Rate of cearance of SG per cell along the sucrose treatment. The curves were calculated based on data obtained from quantifying 3-4 frames of 1,024 × 1,024 pixels. Each experiment was repeated at least twice. The data were analyzed by 2-tailed unpaired t-test  *p < 0.05,  **p < 0.01,  ***p < 0.001.

Osmotic stress, typically induced by high concentrations of NaCl or other osmolytes, effectively triggers the formation of SGs (Protter and Parker, 2016; Hu et al., 2023). To explore the role of CL activity in SG assembly during osmotic stress, we treated cells with NaCl. Unlike arsenite treatment, NaCl-induced SG assembly occurred much faster, reaching its maximal level within 32 min, in both wt and T35A mutant cells. The number of SGs per cell was significantly lower in PSMB6 T35A mutant cells compared to wild-type cells (Figures 4A,B). Interestingly, the size of SGs in the mutant cells increased over time and became significantly larger than those in wild-type cells (Figure 4C). The rate of clearance per cell was faster in PSMB6 T35A cells (Figure 4D).

Next, we induced osmotic stress by treating cells with sucrose. Under this condition, SG formation was slower in PSMB6 T35A mutant cells (Figures 4E,F). The total number of SGs formed was significantly lower in PSMB6 T35A cells (Figure 4F), and the size of SGs was smaller at the early time but increased later on (Figure 4G). The rate of clearance per cell was significantly slower in PSMB6 T35A cells (Figure 4H). These findings suggest that proteasome CL activity is critical for the efficient assembly of SGs under both proteotoxic arsenite stress and osmotic stress conditions. The data highlight the role of CL activity in regulating SG formation and clearance.

### 3.5 Proteasome caspase-like (CL) activity regulates proteasome granule dynamic

**FIGURE 5:**
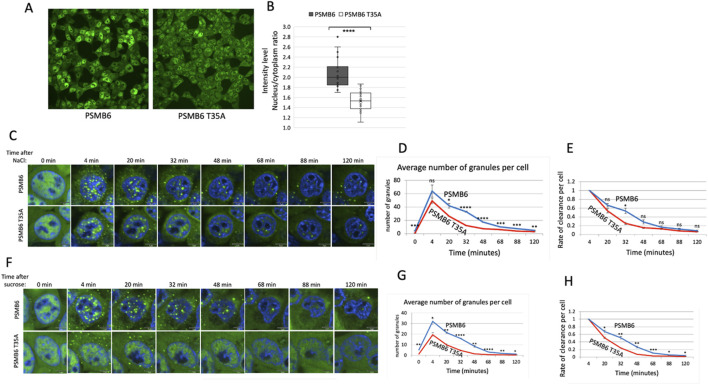
PSMB6 T35A cells exhibit altered nuclear localization and rapid clearance of PGs under osmotic stress. **(A)** Representative images showing subcellular localization of proteasomes in PSMB6 and PSMB6 T35A cells under normal conditions. YFP-tagged PSMB6 displays strong nuclear localization, while PSMB6 T35A exhibits reduced nuclear localization and increased cytoplasmic distribution. **(B)** Quantification of nuclear-to-cytoplasmic intensity ratios of YFP-tagged proteasomes in PSMB6 and PSMB6 T35A cells. Data indicate a significant reduction in nuclear localization in PSMB6 T35A cells (****p < 0.0001). **(C,F)** Time-lapse images of PG formation and clearance following osmotic stress induced by NaCl **(C)** or sucrose **(F)**. Both PSMB6 and PSMB6 T35A cells form PGs within 4 minutes of treatment. **(D,G)** Quantification of the average number of PGs per cell over time after NaCl **(D)** or sucrose **(G)** treatment. PSMB6 T35A cells exhibit significantly fewer PGs at their peak and a faster clearance compared to PSMB6 cells. **(E,H)** Rate of PG clearance per cell following NaCl **(E)** or sucrose **(H)** treatment. PSMB6 T35A cells show a significantly faster PG dissolution. We present the relative levels of PGs over time, with values normalized to 1 at the 4-min time point for both strains. The scale bar represents 5 μm. The curves were calculated based on data obtained from quantifying 3-4 frames of 1,024 × 1,024 pixels. Each experiment was repeated at least twice. (**p < 0.01,  ***p < 0.001).

It has been reported that under osmotic stress, proteasomes rapidly form nuclear granules (condensates) in cells (Yasuda et al., 2020; Steinberger et al., 2023). Proteasome inhibitor MG-132, which specifically inhibits the chymotrypsin-like activity of the β5 subunit, increases the number and size of the proteasome granules and significantly delays their dissolution (Yasuda et al., 2020). Here, we investigated whether CL activity also regulates proteasome granules. To address this, we used CRISPR technology to YFP tag the PSMB6 and PSMB6 T35A genes, as previously described (Reuven et al., 2019; Steinberger et al., 2023). Interestingly, we observed that proteasomes in PSMB6 T35A cells were significantly less localized in the nucleus and showed higher levels in the cytoplasm (Figure 5A). Quantification confirmed a significant reduction in the nuclear-to-cytoplasmic intensity ratio in PSMB6 T35A cells compared to wild-type PSMB6 cells (Figure 5B).

Next, we exposed the cells to osmotic stress using NaCl. As expected, we observed the formation of proteasome granules (PGs) within 4 minutes in both PSMB6 and PSMB6 T35A cells (Figure 5C). However, the number of PGs per cell reached a lower peak in PSMB6 T35A cells. It disassembled more rapidly than PSMB6 cells (Figures 5D,E), suggesting that the mutation impacts the stability of PGs under stress.

Similar results were observed when the cells were subjected to osmotic stress induced by sucrose (Figures 5F–H). In this case, the maximum number of PGs per cell in PSMB6 T35A cells was significantly lower than in wild-type cells. Additionally, the PGs in PSMB6 T35A cells were predominantly localized in the cytoplasm. This cytoplasmic localization and the rapid dissolution of PGs further support the notion that the CL activity is not essential for the initial formation of the PGs but is crucial for maintaining their stability. However, we cannot exclude the possibility that the cytoplasmic PGs are inherently unstable. This highlights a potential role for CL activity in regulating the localization of proteasome condensate and its dynamics under stress conditions.

### 3.6 PSMB6 T35A cells enhance arsenite-induced formation of proteasome dispersed granules

**FIGURE 6:**
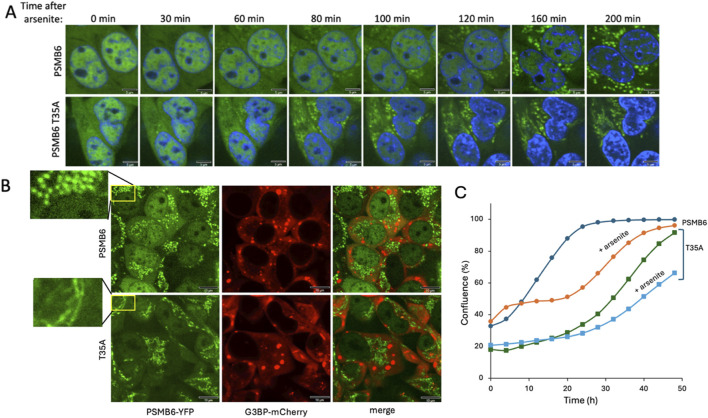
Proteasome granule formation and cell growth dynamics under arsenite-induced stress. **(A)** Time-course analysis of proteasome granule formation in PSMB6 and PSMB6 T35A cells following 3 h s treatment of 1 mM arsenite. PGs (green) appear in the cytoplasm after 80 min in PSMB6 cells and after 60 min in PSMB6 T35A cells, as observed by fluorescence microscopy. Nuclei are stained with DAPI (blue). The scale bar represents 5 µm. **(B)** Simultaneous analysis of PGs (PSMB6-YFP, green) and SGs (G3BP-mCherry, red) in PSMB6 and PSMB6 T35A cells under arsenite treatment. The magnified insets (left) highlight the distinct spatial pattern: punctate and dispersed in wild-type versus cortical or perinuclear clustering in the T35A mutant. No colocalization is observed, indicating that proteasome and stress granules are distinct entities. The scale bar represents 10 µm. **(C)** Growth kinetics of PSMB6 and PSMB6 T35A cells under control and arsenite-treated conditions. Arsenite-treated PSMB6 cells show biphasic growth with recovery after 20 h, whereas arsenite-treated PSMB6 T35A cells exhibit slower growth and fail to recover by 50 h. The growth curve was analyzed *in situ* using the Incucyte® SX1 live-cell analysis system.

The formation of PGs in animal cells in response to osmotic stress has been previously documented (Yasuda et al., 2020; Steinberger et al., 2023). However, whether proteotoxic stress induces PGs formation has not been examined in depth. Earlier studies reported that arsenite treatment does not induce the PGs up to 60 min of treatment, whereas osmotic stresses triggers their formation within a few minutes (Yasuda et al., 2020; Steinberger et al., 2023). In this study, we treated cells with arsenite for a longer duration and observed the formation of PGs after 80 min of treatment. Surprisingly, the PGs are not nuclear but exclusively localized in the cytoplasm (Figure 6A). In control cells, numerous bright, punctate granules are distributed throughout the cytoplasm, indicating robust granule formation. In contrast, PSMB6 T35A mutant cells exhibit fewer and more dispersed granules. These observations suggest that the T35A mutation impairs normal arsenite-induced proteasome granule assembly (Figure 6B). Arsenite is known for not only to trigger protein misfolding but also, destabilize the 26S proteasome (Stanhill et al., 2006), which may underlie the formation of these aberrant PGs, may reflect differences in assembly dynamics or stability in the CL-deficient background. Notably, in PSMB6 T35A mutant cells, the aberrant PG structures appeared earlier—at 60 min after arsenite treatment.

The kinetics of dispersed PGs appearance under arsenite treatment resembles those of SGs in terms of their timing of appearance and cytoplasmic localization. However, simultaneous analysis of both PGs and SGs in the same cells revealed no association between the two types of granules (Figure 6B). This distinction was consistent in both PSMB6 and PSMB6 T35A cells.

Next, we examined the growth dynamics of edited and arsenite-treated cells. Arsenite-treated PSMB6 cells exhibited biphasic growth: their growth rate was slower than untreated cells initially but recovered after 20 h to match the growth rate of untreated cells (Figure 6C). In contrast, untreated PSMB6 T35A cells displayed a slower growth rate than unedited cells but eventually reached confluence after 50 h. However, arsenite-treated PSMB6 T35A cells grew significantly slower and failed to recover after 50 h. These data suggest that arsenite induces cytosolic dispersed PG-like structures and that PSMB6 T35A cells are more susceptible to arsenite stress, showing earlier and morphologically altered granule formation, features that likely contribute to their impaired growth under these conditions.

## 4 Discussion

This study highlights the role of the 20S proteasome’s caspase-like (CL) activity in cellular stress responses. Our findings show that cells devoid of CL activity increase cellular sensitivity to stress, resulting in slower proliferation and heightened vulnerability to arsenite and NaCl-induced stress. The unfolded protein response (UPR) activation in CL-deficient cells, marked by elevated sXBP-1 and stress markers, suggests that CL activity mitigates proteotoxic and genotoxic stress by preventing the accumulation of misfolded or aggregated proteins.

Using *in vitro* 20S proteasome reactions and proteomic approaches, we show that CL activity selectively degrades intrinsically disordered proteins (IDPs) and regions (IDRs) (Myers et al., 2018), which are key components of liquid-liquid phase separation (Wang et al., 2018), such as SGs and proteasome condensates. Cells harboring this mutation exhibited slower SG assembly during arsenite-induced proteotoxic stress and reduced SG stability under osmotic stress. These observations highlight the dual role of CL activity in facilitating both the efficient assembly and the structural integrity of SGs. Furthermore, the rapid dissolution of proteasome condensates in CL-deficient cells underscores the importance of CL activity in sustaining the dynamics of these assemblies under stress conditions. However, we could not rule out the possibility of SG fusion contributing to the observed reduction in granule number given the increase in size. Our analysis focused primarily on granule number and median area over time, and did not directly distinguish between dissolution versus fusion events.

The observation that stress granule (SG) assembly is delayed in cells lacking CL activity is counterintuitive, as reduced degradation of intrinsically disordered proteins (IDPs) might be expected to promote, rather than impair, condensate formation. Furtheremore, the CL mutant cells demonstrate a certain level of proteotoxicity which is expected to trigger SG formation (Mateju et al., 2017). This unexpected finding raises important questions about the underlying molecular mechanisms. It has been reported that SG assembly is driven by interactions among RNA molecules, SG proteins like G3BP1, and homotypic/heterotypic interactions involving IDRs (Kedersha et al., 2016; Panas et al., 2016; Jain and Vale, 2017; Van Treeck and Roy, 2018; Protter et al., 2018). More recently, it has been reported that RNA binding disrupts an autoinhibitory interaction within G3BP1, facilitating SG assembly (Guillén-Boixet et al., 2020). Based on our findings, we propose that CL activity may regulate SG dynamics either through direct processing of SG-associated proteins, such as G3BP1, to relieve autoinhibition, or alternatively—but not mutually exclusively—through the action of peptides generated via proteasomal CL activity that may, through a yet unresolved mechanism, facilitate SG assembly.

Interestingly, while CL activity was not strictly required for the initial formation of proteasome granules under osmotic stress, mutant cells displayed fewer and less stable granules. This unexpected result suggests a potential indirect role for CL activity, linked to stress-induced changes in CL-deficient cells. Alternatively, it raises the intriguing possibility of a direct role for CL activity in granule formation, perhaps by processing essential components or regulators. Additionally, the acidic peptides generated by CL activity may directly contribute to proteasome granule formation, warranting further investigation.

Under inflammation, animal cells express three proteasomal proteolytically active subunits: β1i, β2i, and β5i, collectively termed immunosubunits due to their cytokine-inducible expression and role in antigenic peptide generation (Schmidt and Finley, 2014; Nathan et al., 2013; Seifert et al., 2010). These subunits replace the constitutive proteasome subunits β1, β2, and β5. β1i provides chymotrypsin-like activity, replacing the caspase-like activity of β1, rendering the immunoproteasome’s caspase-like activity nearly nonfunctional. Whether the unique behavior of cells lacking caspase-like activity is relevant under inflammation signaling remains an open question.

Our findings highlight the multifaceted role of the 20S proteasome’s CL activity in maintaining proteostasis under stress conditions. Possibly by targeting IDP/IDR-enriched substrates, this activity regulates SG dynamics and proteasome granules stability, processes essential for cellular adaptation to stress (Li et al., 2013; Markmiller et al., 2018; Kedersha and Paul, 2009). Future studies should focus on elucidating the molecular mechanisms underlying the selective targeting by CL activity and its broader implications in diseases associated with proteostasis imbalances, such as neurodegenerative disorders and cancer.

## Data Availability

The raw data supporting the conclusions of this article will be made available by the authors, without undue reservation.
